# Geographical distribution of the dispersal ability of alien plant species in China and its socio-climatic control factors

**DOI:** 10.1038/s41598-021-85934-8

**Published:** 2021-03-30

**Authors:** Quanlai Zhou, Jing Wu, Xue Cui, Xuehua Li, Zhimin Liu, Ala Musa, Qun Ma, Haibin Yu, Wei Liang, Shaoyan Jiang, Yongcui Wang

**Affiliations:** 1 CAS Key Laboratory of Forest Ecology and Management, Institute of Applied Ecology, Chinese Academy of Sciences, Shenyang, 110016 China; 2 Institute of Applied Ecology, Chinese Academy of Sciences, 72 Wenhua Road, Shenyang, 110016 Liaoning Province China; 3 Taizhou University, Taizhou, 384000 Zhejiang Province China; 4 Station of Forest and Grassland Pest Control and Quarantine of Liaoning Province, Shenyang, 110804 China; 5 Liaoning Vocational College of Ecological Engineering, Shenyang, 110101 China

**Keywords:** Ecology, Invasive species

## Abstract

Dispersal ability is important for the introduction, establishment, and spread of alien plant species. Therefore, determination of the geographical distribution of the dispersal ability of such species, and the relationship between dispersal ability and socio-climatic factors are essential to elucidate the invasion strategies of the alien plant species. Analytic hierarchy process and inventory, risk rank, and dispersal mode data available on Chinese alien plant species were used to determine their dispersal ability, the geographical distribution thereof, and the relationship between socio-climatic factors and dispersal ability. High-risk alien plant species had a higher natural dispersal ability (or several natural dispersal modes) but a lower anthropogenic dispersal ability (or few anthropogenic dispersal modes) than low-risk alien plant species. The geographical distribution of the dispersal ability of the alien plant species showed an inverse relationship with species density. Alien plant species with low dispersal ability (i.e., with fewer dispersal modes and distribution in the southeast) showed a tendency to adapt to environments with mild climates, while those with high dispersal ability (i.e., with more disposal nodes and distribution in the northwest) showed a tendency to adapt to harsh environments. It is essential for land managers and policy makers to understand the geographical distribution of the dispersal ability of alien plant species and their socio-climatic control factors to formulate strategies to control the natural and anthropogenic dispersal of such plants.

## Introduction

Alien plant species can considerably threaten biodiversity and stability of ecosystems; change their structure, function, and biogeographic distribution; and eventually cause environmental degradation and substantial socioeconomic damage^1–6^. Increases in urbanization and the human population are often accompanied by unprecedented ecosystem alteration and modification. With the rapid expansion of international trade, tourism, and transportation, alien plant species can invade ecosystems beyond their natural range, establish communities, and threaten native biodiversity^3,6–8^.

Diaspores of several alien plant species have successfully developed specialized morphological structures for multiple dispersal modes, enabling them to use both biotic and abiotic natural dispersal agents arriving in a new environment^9–13^. Dispersal modes are critical determinants of the invasion rate at which alien plant species occupy new habitats^13,14^. Dispersal modes associated with wind, water, animals, and other plants can be categorized as natural strategies (those performed without human involvement)^10,15–21^, while those associated with human activities are categorized as anthropogenic strategies^13,22,23^. Alien plant species, particularly rapid dispersers, usually use both strategies^14^.

The ability of alien plant species to extend their diaspore dispersal distance to the maximum possible extent may be critical to their invasion, survival, and expansion in a new environment^14,24^. Diaspore dispersal distance, which depends on both plant traits and environmental conditions, should therefore be an important variable representing the ability to extend dispersal distance^15,19,20,25–28^. However, it is difficult to monitor the dispersal distance of plants via field studies^13,27,29^, particularly for those that have different dispersal modes, because each mode corresponds to a particular dispersal distance^15,20,22,23,27,28^. For example, a diaspore can use more than one dispersal mode based on its morphological characteristics. Species that are dispersed by wind (anemochory) can present with the following five types of dispersal modes based on their morphology: 1) chamaechory (an entire round plant that rolls on the ground); 2) cystometeorochory (balloon-like diaspores that glide or roll on the ground); 3) trichometeorochory (diaspores with pappi and that float in the air); 4) pterometeorochory (diaspores with wings and descend in a spiral); and 5) semachory (small diaspores with no special morphology and float in the air)^10,13,15,20,30^. Therefore, the dispersal mode–a combination of dispersal agents, plant traits, and environmental conditions–represents a considerable component of the ability of plants to extend their dispersal distance^15^.

Several ranks of the risk from alien plant species were utilized to meet the requirements of policy makers who were responsible for developing strategies to manage invasive alien plants in China^3,18,31–35^. Ma et al.^35^ have reported that in China, alien plant species are classified into the following six risk ranks, from high- to low-risk, based on their biological traits, scale of possible invasion, and socioeconomic damages caused: I) malignant invaders, II) serious invaders, III) local invaders, IV) mild invaders, V) species requiring further observation, and VI) cultivated alien species. The ability of alien plant species in different risk ranks to extend their dispersal distance is important for invasion control^18^. Further studies should also be conducted to assess the dispersal ability of alien plant species that are in different risk ranks and those that use both natural and anthropogenic dispersal agents.

China spans across four precipitation regimes (humid, semi-humid, semi-arid, and arid) from east to west because of its remarkable geographical diversity and continental monsoon climate. From north to south, the country also covers five climatic zones (cold temperate, temperate, warm temperate, subtropical, and tropical)^36,37^. Additionally, regional population density and the gross domestic product (GDP) tend to decrease from southeast to northwest^38,39^. Therefore, certain socioeconomic factors associated with population may be closely related to the dispersal ability of alien plant species. Previous studies have shown that the density of alien plant species decreases from the southeast coast to the northwest inland region of China^2,3,11,40–42^. Factors influencing this regional distribution pattern of alien plant species density (or richness) can be natural (e.g., precipitation and temperature) and/or anthropogenic (e.g., density of human population, GDP, and GDP per capita)^2,11,42^. Therefore, both natural and anthropogenic factors should be considered as influencing factors that impact the regional distribution of the dispersal ability of alien plant species.

In this study, we used an inventory, risk ranks, and information of the dispersal modes of alien plant species to examine their dispersal ability, geographical distribution, and the relationship between socio-climatic factors and dispersal ability. Our objectives were to determine the following: 1) the characteristics of alien plant species’ dispersal abilities, including those in different risk ranks, 2) the geographical distribution of the dispersal abilities across China, and 3) the relationships between socio-climatic factors and dispersal abilities. The results found based on these objectives might help land managers and policy makers to control alien plant species in China.

## Methods

### Data collection

Data on 562 species of alien plants and their distribution in China were collected based on *The Checklist of the Chinese Invasive Plants*^35^. The alien plant species were distributed in 34 provincial-level administrative regions–twenty-three provinces, five autonomous regions, four municipalities, and two special administrative regions^2,11,35,43,44^. We collected the data on morphological traits of diaspores from several sources, including the Flora of China (http://www.iplant.cn/foc/), the PLANTS database managed by the United States Department of Agriculture (https://plants.usda.gov/java/), and the Seed Information Database from the Royal Botanic Gardens, Kew (http://data.kew.org/sid/). We retrieved references from the China National Knowledge Infrastructure database (CNKI, https://www.cnki.net/), Baidu (https://www.baidu.com/), and Web of Science (http://apps.webofknowledge.com) using the Latin name of the alien plant species as our keywords, and reviewed them to obtain information related to dispersal modes.

### Definition of risk ranks for alien plant species

The alien plant species in China were categorized into six ranks, from high- to low-risk, based on their biological and ecological characteristics, distribution, and influence on ecosystems and the national economy (Table 1)^35,41,45,46^.

**Table 1:** Definitions of the six risk ranks and the percentage of alien plant species in China that belonged in each.

Rank	Description	Category^46^	Invasion scale	Distribution	Loss	Number of species	Percentage (%)
I	Malignant invaders	Transformer	National scale	More than one physical geographical region	Enormous economic and ecological loss	37	6.4
II	Serious invaders	Invasive	National scale	At least one physical geographical region	Enormous economic and ecological loss and a bad influence on society	50	9.0
III	Local invaders	Invasive	Regional scale	At least one physical geographical region	Big economic and ecological loss	73	13.3
IV	Mild invaders	Invasive	Local or national scale	Unable to invade into new geographical regions	Low economic and ecological loss	79	14.2
V	Requiring further observation	Naturalized non-invasive	Unknown	Undetermined	In a naturalized state	225	39.8
VI	Cultivated alien species	Cultivated	National scale	Under cultivation	No loss	98	17.3
Total						562	100

### Selection of socio-climatic factors and collection of data

We selected the four climatic factors–mean annual humidity (MAH), mean annual precipitation (MAP), mean annual temperature (MAT), and mean frost days (MFD)–to examine the relationship between them and determined the dispersal ability of alien plant species^44^. The climatic data were obtained from a dataset containing information from 2,086 ground meteorological stations in China for the period 1981 to 2010 (http://data.cma.cn/)^44^. The annual mean values for each climatic factor were obtained by collecting monthly data from these stations, which were distributed in the 34 provincial-level administrative regions.

We collected data on the GDP, population density, and GDP per capita (GPC, i.e., GDP/population) from the *Statistical Database of China’s Economic and Social Development* (http://data.cnki.net/) for the period 2009 to 2018^44,47^.

### Diaspore dispersal modes used by alien plant species

**Figure 1:**
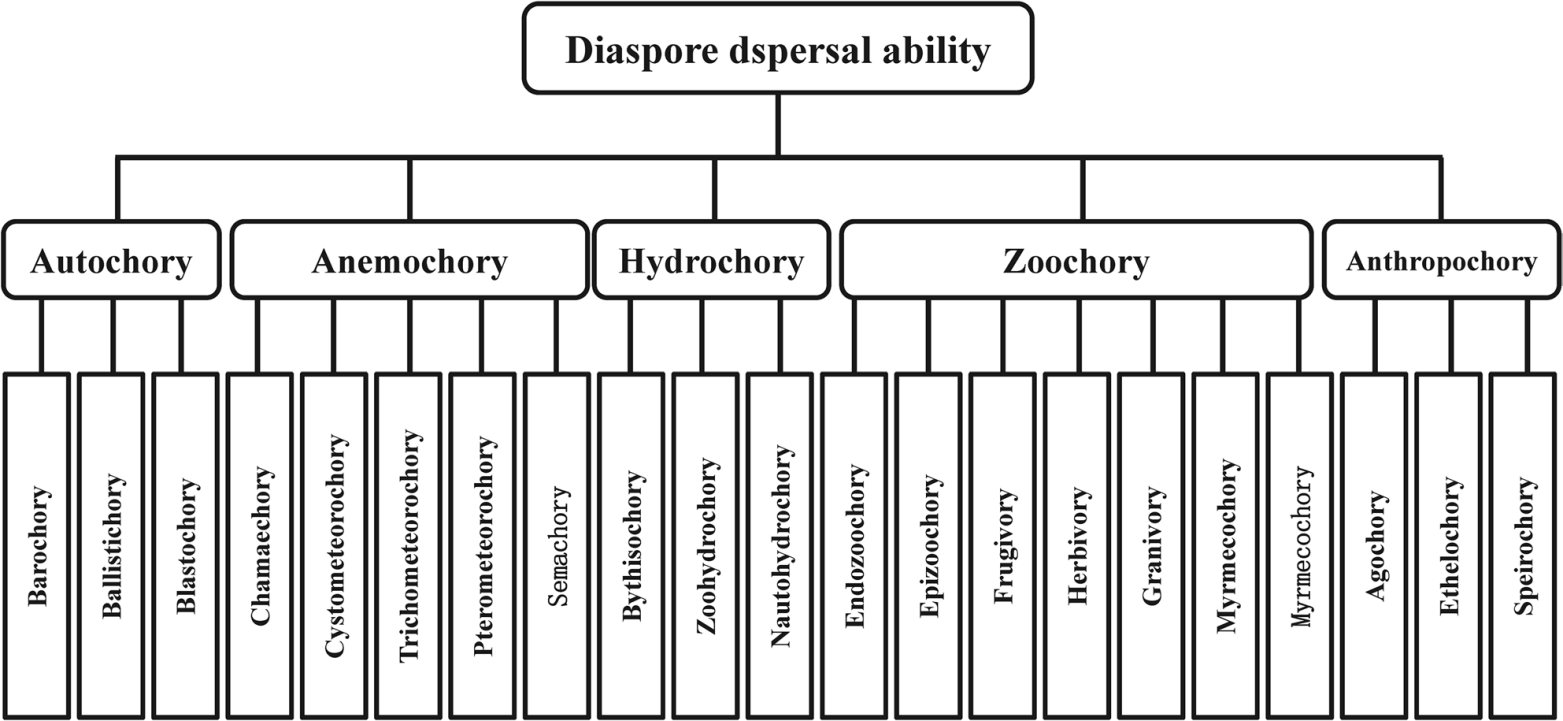
Five dispersal strategies with twenty-one dispersal modes.

Diaspores often present with multiple dispersal modes to enable spread via different agents^14,16^. We listed 21 dispersal modes associated with the following 5 dispersal strategies (i.e., 5 dispersal vectors): gravity or the plant itself (autochory), wind (anemochory), water (hydrochory), wild or domestic animals (zoochory), and humans (anthropochory) (Fig. 1). Detailed descriptions are mentioned in the Supplementary Materials)^10,15–17^.

We classified autochory, anemochory, hydrochory, and zoochory as natural dispersal modes and anthropochory as an anthropogenic dispersal mode (Fig. 1); detailed descriptions are mentioned in Supplementary Material 1.

### Evaluation of diaspore dispersal ability

In this study, we defined the ability of alien plant species to extend their diaspore dispersal distance as dispersal ability without considering the roles of the number of diaspores dispersed due to lack of data on the latter. To overcome selective pressures arising from both biotic and abiotic factors, plant species rely on multiple dispersal modes to facilitate the spread of their diaspores^14^. Diaspore dispersal ability can be used to estimate the dispersal modes used^15^; therefore, the dispersal ability of a species is a consequence of a combination of multiple dispersal modes. Using the technique of analytic hierarchy process (AHP), we first assigned a weight to each dispersal strategy and mode based on the dispersal distance they corresponded to by calculating the relative importance of two strategies and modes and by generating a pairwise comparison matrix of the dispersal strategies and modes in each of these (Supplementary Material 2). We then determined the dispersal ability of a species by adding the scores of the multiple dispersal modes used (Supplementary Material 2).

### Data analysis

We conducted regression analyses to determine the influence of socio-climatic factors on diaspore dispersal ability. Parameter fitting of the regressions and analysis of variance (ANOVA) and Duncan’s tests for multiple comparisons of dispersal ability were conducted (with statistical significance as *p* < 0.05 for Duncan’s test) using the software PASW Statistics 18.0 (IBM Inc., Armonk, New York, USA). Histograms of the changes in dispersal ability and the distribution of alien plant species at different risk ranks were illustrated using SigmaPlot 10.0 (Systat Software, Inc., San Jose, California, USA). The software Matlab (MathWorks Inc. Natick, Massachusetts, USA) was used to perform tests using the AHP to determine the dispersal modes and to evaluate the dispersal ability of alien plant species. We used ArcMap 10 (ESRI Inc., Redlands, California, USA) to generate maps illustrating the distribution of the dispersal abilities of the alien plant species and of the different risk ranks observed in 34 provincial-level administrative regions in China.

## Results

### Dispersal ability of alien plant species in six risk ranks

**Figure 2:**
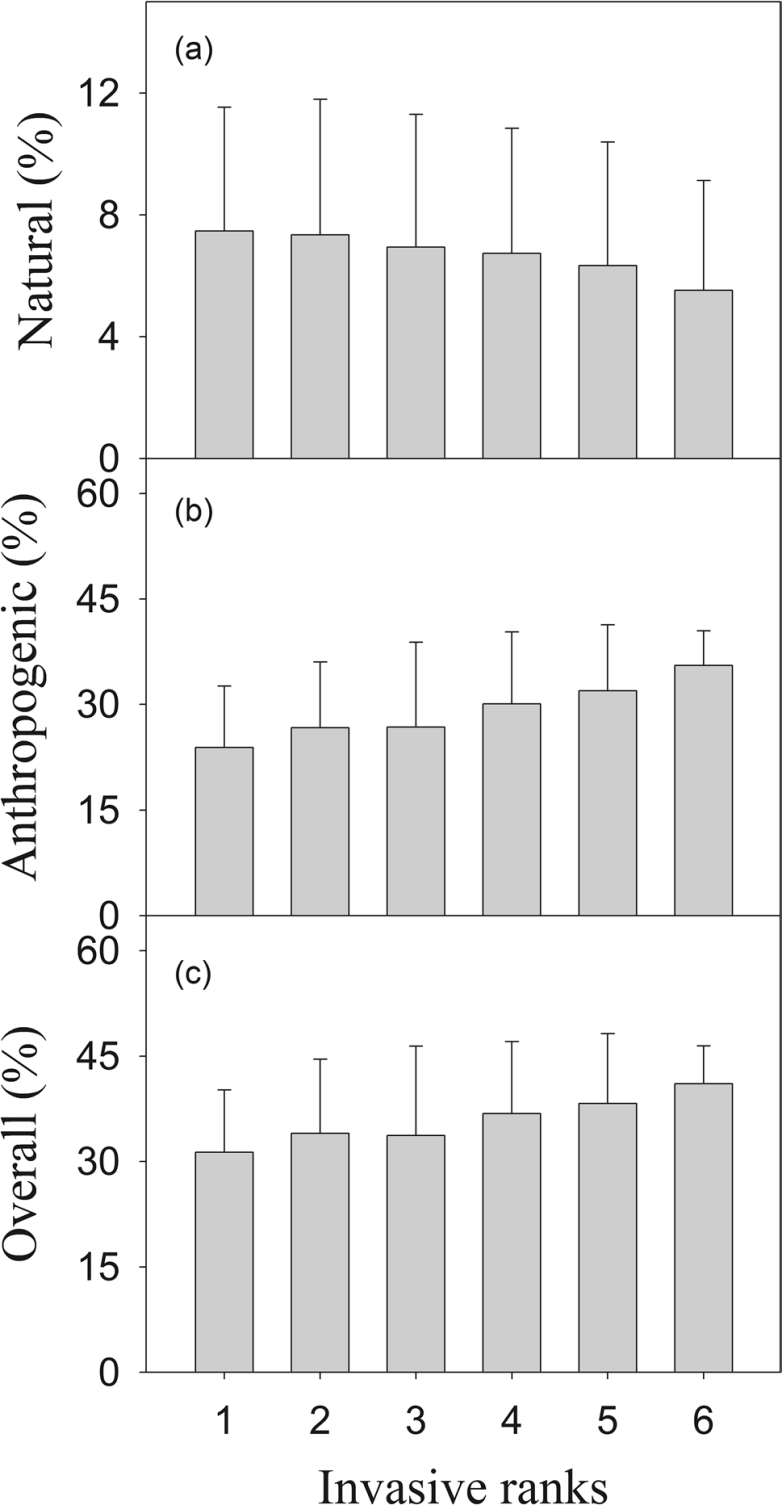
Dispersal ability of the alien plant species across China in six risk ranks. (**a**) Natural dispersal ability; (**b**) anthropogenic dispersal ability; (**c**) overall dispersal ability. (**I**) Rank I: malignant invaders; (**II**) rank II: serious invaders; (**III**) rank III: local invaders; (**IV**) rank IV: mild invaders; (**V**) rank V: species requiring further observation; and (**VI**) rank VI: cultivated alien species. The plot was drawn by SigmaPlot 10.0 (https://systat-sigmaplot.com).

Our results showed that there was a significant decreasing trend (*p* < 0.05) in the natural dispersal ability, but an increasing trend (*p* < 0.01) was observed in the anthropogenic dispersal ability for plants in ranks I to VI (Fig. 2, Table 2). Overall, there was an increase (*p* < 0.01) in both natural and anthropogenic dispersal abilities for plants in rank I to VI (Fig. 2, Table 2).

**Table 2:** ANOVA results for the natural, anthropogenic, and overall natural and anthropogenic dispersal abilities of alien plant species in the six risk ranks.

Source of variation		d.f	MS	*F*	*P*
Natural	Risk ranks	5	0.004	2.339	0.041
dispersal ability	Error	556	0.002		
Anthropogenic	Risk ranks	5	0.193	14.184	0.000
Dispersal ability	Error	556	0.014		
Overall	Risk ranks	5	0.142	9.038	0.000
	Error	556	0.016		

### Geographical distribution of the dispersal ability of the alien plant species

**Figure 3:**
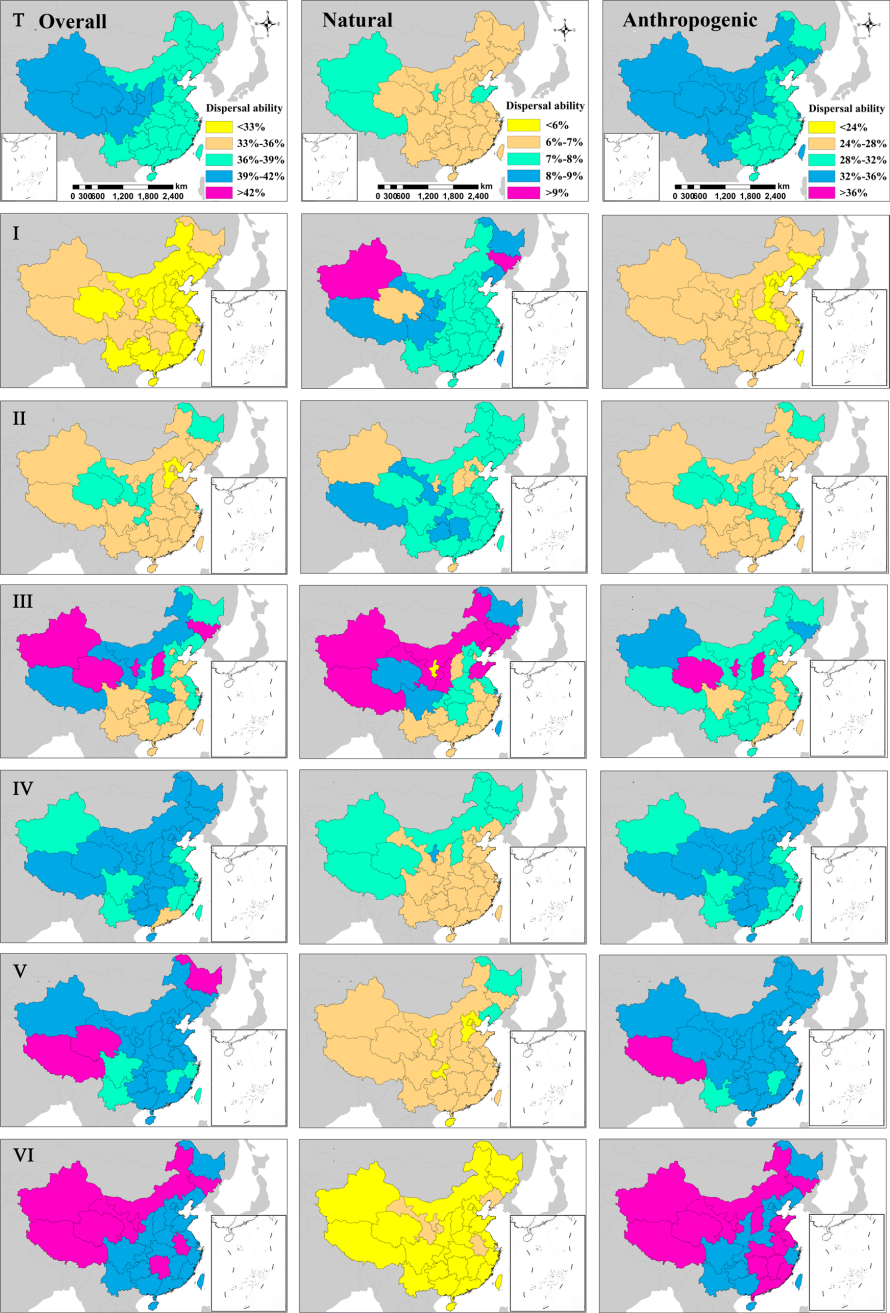
Geographical distribution of dispersal ability of all species studied and six risk ranks (**I**–**VI**) for the overall, natural, and anthropogenic dispersal abilities across China. (T) Total alien plant species; (**I**) rank I: malignant invaders; (**II**) rank II: serious invaders; (**III**) rank III; local invaders; (**IV**) rank IV; mild invaders; (**V**) rank V; species requiring further observation; (**VI**) rank VI: cultivated alien species. Maps generated by ArcMap 10.0 (https://support.esri.com/zh-cn/Products/Desktop/arcgis-desktop/arcmap/10).

The overall, natural, and anthropogenic dispersal ability of all alien plant species showed an increasing trend from southeast to northwest regions of China (Fig. 3 Ta, Tb, Tc). The variations in dispersal ability (i.e., the differences between the highest and lowest dispersal abilities) for all alien plant species within the 34 administrative regions studied were 3.0%, 1.5%, and 2.7%, respectively, overall and in natural and anthropogenic dispersal modes (Fig. 3 Ta, Tb, Tc). In terms of the risk ranks, the variations in overall dispersal ability were 4.6%, 5.1%, 13.2%, 5.4%, 4.6%, and 3.0% for plants in ranks I, II, III, IV, V, and VI, respectively. The variations in natural dispersal ability were 3.3%, 1.9%, 7.1%, 2.1%, 1.7%, and 1.4%, respectively, for plants in ranks I, II, III, IV, V, and VI. Finally, the variations in anthropogenic dispersal ability were 4.8%, 3.9%, 13.5%, 4.9%, 5.3%, and 2.5% for plants in ranks I, II, III, IV, V, and VI, respectively.

### Relationships between the dispersal ability and socio-climatic factors

The overall, natural, and anthropogenic dispersal ability of all alien plant species showed significantly negative relationships with GDP–with *p* < 0.01 and *p* < 0.05 for overall and natural dispersal ability, respectively–and MAH (Table 3). Natural dispersal ability was positively correlated with MAP (*p* < 0.01) for plants in rank II, MFD (*p* < 0.01) and MAT (*p* < 0.05) for those in rank III, and MFD (*p* < 0.01) for plants in rank V; however, it was negatively correlated with MAH (*p* < 0.01) for plants in rank IV. Anthropogenic dispersal ability was positively correlated with MAP (*p* < 0.01) for plants in rank I but negatively correlated with MAP (*p* < 0.01), MAH (*p* < 0.01), MFD (*p* < 0.05), and MAH (*p* < 0.01) for plants in ranks III, IV, V, and VI, respectively (Table 3). Overall dispersal ability was positively correlated with GDP (*p* < 0.01) and MFD (p < 0.01) for plants in ranks I and V, respectively, and negatively correlated with MAH for plants in rank III at p < 0.01; GDP for plants in ranks IV and V at *p* < 0.05 and *p* < 0.01, respectively; and MAH for plants in rank VI at *p* < 0.01 (Table 3).

**Table 3:** ANOVA analysis and estimation of regression coefficients among the natural, anthropogenic, and overall dispersal abilities and socio-climatic factors for all alien plant species and each of the six risk ranks thereof.

Rank	ANOVA							Coefficient			
	Dispersal ability	R^2^	Model	d.f	MS	F	P	Independent	B	t	P
Total	Natural	0.44	Regression	1	1.2	24.7	0.000	Intercept	7.9	30.4	0.000
			Error	32	0.05			MAH	− 0.02	− 5.0	0.000
	Anthropogenic	0.15	Regression	1	2.4	5.7	0.023	Intercept	32.3	186.8	0.000
			Error	32	0.4			GDP	− 0.11	− 2.4	0.023
	Overall	0.30	Regression	1	6.3	14.0	0.001	Intercept	41.7	51.4	0.000
			Error	32	0.5			GDP	− 0.04	− 3.7	0.001
I	Natural	–	–	–	–	–	–	–	–	–	–
	Anthropogenic	0.26	Regression	1	9.5	11.2	0.002	Intercept	23.6	70.9	0.000
			Error	32	0.9			MAP	0.01	3.3	0.002
	Overall	0.31	Regression	1	12.5	14.2	0.001	Intercept	31.2	92.6	0.000
			Error	32	0.9			GDP	0.001	3.8	0.001
II	Natural	0.235	Regression	1	1.6	9.8	0.004	Intercept	7.1	48.5	0.000
			Error	32	0.2			MAP	0.00	3.1	0.004
	Anthropogenic	–	–	–	–	–	–	–	–	–	–
	Overall	–	–	–	–	–	–	–	–	–	–
III	Natural	0.54	Regression	2	18.8	18.6	0.000	Intercept	5.4	10.6	0.000
			Error	31	1.0			MFD	0.03	5.8	0.000
								MAT	0.07	2.3	0.025
	Anthropogenic	0.24	Regression	1	78.7	10.3	0.003	Intercept	40.4	12.1	0.000
			Error	32	7.6			MAP	− 0.15	− 3.2	0.003
	Overall	0.46	Regression	1	181.2	27.8	0.000	Intercept	53.6	17.4	0.000
			Error	32	6.5			MAH	− 0.23	− 5.3	0.000
IV	Natural	0.4	Regression	1	2.5	21.3	0.000	Intercept	8.7	20.9	0.000
			Error	32	0.1			MAH	− 0.03	− 4.6	0.000
	Anthropogenic	0.28	Regression	1	14.5	12.7	0.001	Intercept	33.5	117.7	0.000
			Error	32	1.1			MAH	− 0.27	− 3.6	0.001
	Overall	0.48	Regression	2	15.2	14.1	0.000	Intercept	44.3	35.2	0.000
			Error	31	1.1			GDP	− 0.25	− 3.2	0.003
								MAH	− 0.06	− 3.0	0.004
V	Natural	0.44	Regression	2	0.95	12.2	0.000	Intercept	3.7	5.6	0.000
			Error	31	0.08			MFD	0.01	4.9	0.000
								MAH	0.03	3.7	0.001
	Anthropogenic	0.179	Regression	1	7.6	7.0	0.013	Intercept	34.6	123.9	0.000
			Error	32	1.1			MFD	− 0.20	− 2.6	0.013
	Overall	0.38	Regression	2	8.6	9.3	0.001	Intercept	40.1	108.3	0.000
			Error	31	0.9			MFD	0.02	3.0	0.005
								GDP	− 0.16	− 2.3	0.029
VI	Natural	−	−	−	−	−	−	−	−	−	−
	Anthropogenic	0.28	Regression	1	3.1	12.4	0.001	Intercept	38.2	63.7	0.000
			Error	32	0.2			MAH	− 0.03	− 3.5	0.001
	Overall	0.2	Regression	1	3.6	8.1	0.008	Intercept	44.1	54.4	0.000
			Error	32	0.4			MAH	− 0.03	− 2.8	0.008

## Discussion

### Changes in the dispersal ability of alien plant species in six risk ranks

Our results showed that the natural dispersal ability of alien plant species in ranks I to VI exhibited a decreasing trend; however, the total and anthropogenic dispersal abilities showed increasing trends (Fig. 2). These results implied that high-risk alien plant species (i.e., those that resulted in considerable economic and ecological loss) had a higher natural dispersal ability, and low-risk alien plant species (i.e., those that resulted in less or negligible economic and ecological loss) exhibited a lower natural dispersal ability. Possible reasons for this include the fact that high-risk alien plant species rely more on natural dispersal strategies, and rely less on anthropogenic strategies apart from accidental introduction (e.g., speirochory or introduced involuntarily via mixing with grain or fodder)^14,35,48,49^. For example, *Ageratina adenophora* (common name: crofton weed; rank I), which was accidentally introduced to China from Mexico, is a notorious weed that exerts considerable ecological impacts and causes substantial economic losses^50,51^. The species exhibits three natural dispersal modes–trichometeorochory, hydrochory, and zoochory–and two anthropogenic dispersal modes, agochory and speirochory (Supplementary Material 1 and 2)^14,35^. In contrast, cultivated alien species–which are widely introduced, domesticated, and cultivated by humans for applications in food, fodder, medicine, and building and gardening materials–rely more on anthropogenic strategies^11,37,52^. For example, *Mangifera indica* (common name: mango; rank VI) is a nutritious and popular fruit that originates mainly from the Indian subcontinent^53^. The species has two natural dispersal modes–barochory and frugivory–and three anthropogenic dispersal modes, namely agochory, ethelochory, and speirochory (Supplementary Material 1)^35,54^.

### Geographical characteristics of the distribution of the dispersal ability of the alien plant species

The overall, natural, and anthropogenic dispersal ability of all alien plant species in the six risk ranks showed an increasing trend from the southeast to the northwest regions of China (Fig. 3). The geographical distribution of dispersal ability of the alien plant species was inversely related to the number and density thereof^2,3,11,40–42^. The majority of alien plant species with high dispersal ability were distributed in inner areas of the northwest parts of the country; however, the majority of alien plant species with low dispersal ability were distributed in the southeast region. The dispersal ability of alien plant species is considerably dependent on the number and relative importance of each dispersal mode (Supplementary Material 2). Most alien plant species present with at least one anthropogenic dispersal mode^13,23,45,55,56^, although those with a high dispersal ability may possess more natural dispersal modes than those with a low dispersal ability^14^. Therefore, alien plant species distributed in the inner areas of northwest China often present with more dispersal modes than those in the southeast.

### Relationships between socio-climatic factors and dispersal ability

Dispersal ability was negatively correlated with socio-climatic factors (Table 3). Southeast regions of China demonstrate the presence of a mild climate, with high precipitation levels, temperatures, and humidity levels, and this supports the survival of a high density of alien plant species^3,41^. In contrast, northwest regions of China demonstrate the presence of a harsh environment–with low precipitation levels, temperatures, and humidity levels–and therefore supports the survival of a low density of alien plant species^40,41^. However, our findings are contrary to these relationships observed between alien plant species density and socio-climatic factors^2,3,11,40–42^. Our results implied that alien plant species with a low dispersal ability (or fewer dispersal modes) could adapt to mild climatic environments as these might render easier invasion^11^. However, alien plant species with a high dispersal ability (or a greater number of dispersal modes) tend to adapt better to harsh environments and can thus invade these regions successfully^3^. Therefore, studies should be conducted more on the investigation of the northwest regions of China compared to the southeast regions of China in terms of formulation of strategies to prevent the establishment of invasive species. Land managers and policy makers should develop more such measures to eliminate natural dispersal routes of alien plant species, based on their natural dispersal modes, and enforce stricter regulations to control anthropogenic dispersal routes.

This study has an important limitation. Traditionally, researchers have assumed that the diaspore of a plant species uses one dispersal mode that is closely associated to its morphology^10^. This perspective ignores the probability that such diaspores can be dispersed via other dispersal modes. However, the data on the probabilities of this occurrence are unknown for alien plant species in China. Therefore, using the technique of AHP to determine the dispersal ability of the latter by weighting all dispersal modes can reflect a trend in their dispersal ability; however, application of such an approach cannot provide an understanding of the exact mechanism by which such alien plant species cause dispersal of their diaspores.

## Summary

High-risk alien plant species had a higher natural dispersal ability (or more natural dispersal modes) but a lower anthropogenic dispersal ability (or fewer anthropogenic dispersal modes) than low-risk alien plant species. The dispersal ability of alien plant species increased from the southeast to the northwest regions across China, which was contrary to the geographical distribution of species density. Alien plant species with a high dispersal ability possess more dispersal modes than those with a low dispersal ability. Alien plant species with a low dispersal ability (or fewer dispersal modes) can adapt to mild environments; however, alien plant species with a high dispersal ability (or more dispersal modes) can adapt to harsh environments. Understanding the characteristics of the geographical distribution of the dispersal abilities of alien plant species and invasion strategies adopted to achieve dispersal is essential to enable land managers and policy makers to develop preventive measures for the elimination of natural dispersal routes and for enforcement of laws/policies to control anthropogenic dispersal routes.

## Supplementary Information

Supplementary Material 1

Supplementary Material 2

Supplementary Material 3
