# Supplementary material for: Geographical distribution of the dispersal ability of alien plant species in China and its socio-climatic control factors
Source: Sci Rep. 2021 Mar 30;11:7187. doi: 10.1038/s41598-021-85934-8 (PMC8009951; doi:10.1038/s41598-021-85934-8)
Supplement: Supplementary file 1 — Supplementary Material 1 [file 41598_2021_85934_MOESM1_ESM.docx]

**Supplementary material 1**

**Table 1** Twenty one diaspores dispersal modes in five dispersal strategies based on previous literatures

| Dispersal strategies | Dispersal modes | | Description | Reference |
| --- | --- | --- | --- | --- |
| Autochory^*^ | 1 | Barochory | Diaspores fallling from the plant | (van Oudtshoorn & van Rooyen, 1999; Vittoz & Engler, 2007) |
|  | 2 | Ballistichory | Diaspores ejected by explosion |  |
|  | 3 | Blastochory | Diaspores deposited by the stem of the plant growing or crawling on the ground as far as possible from the mother plant |  |
| Anemochory | 4 | Chamaechory | Diaspores (more or less the whole plant) rolling on the ground pushed by wind. | (Guitian & Sanchez, 1992; van Oudtshoorn & van Rooyen, 1999; Vittoz & Engler, 2007) |
|  | 5 | Cystometeorochory | Balloon-like diaspores gliding or rolling by wind |  |
|  | 6 | Trichometeorochory | Diaspores with hairy structure (e.g. pappus) dispersed by wind |  |
|  | 7 | Pterometeorochory | Diaspore with wings dispersed by wind |  |
|  | 8 | Semachory | Diaspore with no special morphology for dispersal by wind |  |
| Hydrochory | 9 | Bythisochory | Semi-floating and non-floating diaspores dispersed by water currents or heavy rains on the bottom of a channel. | (van Oudtshoorn & van Rooyen, 1999; Nilsson *et al.*, 2010) |
|  | 10 | Zoohydrochory | Diaspores despersed by animals, humans and their vessels, moving in the water |  |
|  | 11 | Nautohydrochory | Diaspores despersed by floating and moving on rivers, lakes or ponds |  |
| Zoochory | 12 | Endozoochory | Diaspores eaten by animals and undamaged through their gut | (Culver & Beattie, 1978; Brown *et al.*, 1979; Wheelwright & Orians, 1982; Bernays, 1990; van Oudtshoorn & van Rooyen, 1999; Vittoz & Engler, 2007) |
|  | 13 | Epizoochory | Diaspores transported by animals in fur |  |
|  | 14 | Frugivory | Diaspores dispersed by frugivore as a food resource |  |
|  | 15 | Herbivory | Diaspores dispersed by herbivore |  |
|  | 16 | Granivory | Diaspores dispersed by granivore |  |
|  | 17 | Myrmecochory | Diaspores dispersed by ants. |  |
|  | 18 | Dyszoochory | Diaspores destroyed (eaten and digested) by an animal, but some of the seeds are dropped by accident. |  |
| Anthropochory | 19 | Agochory | Diaspores travelling hidden in goods, cars, soil under soles, with hay, etc. | (van Oudtshoorn & van Rooyen, 1999; Vittoz & Engler, 2007) |
|  | 20 | Ethelochory | Plants or diaspores being sold for agriculture and gardening |  |
|  | 21 | Speirochory | Diaspores being involuntarily mixed with the previous ones |  |

**^*^**Autochory: diaspores dispersed without the help of an external agent

Anemochory: diaspores dispersed by wind

Hydrochory: diaspores dispersed by water

Zoochory: diaspores dispersed by animals, either internally or externally or by caching

Anthropochory: diaspores dispersed by humans

Reference

Bernays, E.A. (1990) Plant secondary compounds deterrent but not toxic to the grass specialist acridid Locusta migratoria: Implications for the evolution of graminivory. *Entomologia Experimentalis Et Applicata*, **54**, 53-56.

Brown, J.H., Reichman, O.J. & Davidson, D.W. (1979) Granivory in desert ecosystems. *Annual Review of Ecology and Systematics*, **10**, 201-227.

Culver, D.C. & Beattie, A.J. (1978) Myrmecochory in Viola - dynamics of seed-ant interactions in some west-Virginia species. *Journal of Ecology*, **66**, 53-72.

Guitian, J. & Sanchez, J.M. (1992) Seed dispersal spectra of plant-communities in the Iberian Peninsula. *Vegetatio*, **98**, 157-164.

Nilsson, C., Brown, R.L., Jansson, R. & Merritt, D.M. (2010) The role of hydrochory in structuring riparian and wetland vegetation. *Biological Reviews*, **85**, 837-858.

van Oudtshoorn, K.v.R. & van Rooyen, M.W. (1999) *Dispersal biology of desert plants*. Springer Berlin Heidelberg.

Vittoz, P. & Engler, R. (2007) Seed dispersal distances: a typology based on dispersal modes and plant traits. *Botanica Helvetica*, **117**, 109-124.

Wheelwright, N.T. & Orians, G.H. (1982) Seed dispersal by animals: Contrasts with pollen dispersal, problems of terminology, and constraints on coevolution. *American Naturalist*, **119**, 402-413.
