# Supplementary material for: Geographical distribution of the dispersal ability of alien plant species in China and its socio-climatic control factors
Source: Sci Rep. 2021 Mar 30;11:7187. doi: 10.1038/s41598-021-85934-8 (PMC8009951; doi:10.1038/s41598-021-85934-8)
Supplement: Supplementary file 2 — Supplementary Material 2 [file 41598_2021_85934_MOESM2_ESM.docx]

**Supplementary material 2**

Using AHP method to evaluate diaspore dispersal ability in eight steps (Fig. 1)

Fig. 1 Steps of analytic hierarchy process (AHP) method for evaluation of diaspore dispersal ability

Step 1. Determine the aim, dispersal strategies and dispersal modes (Figure 2).


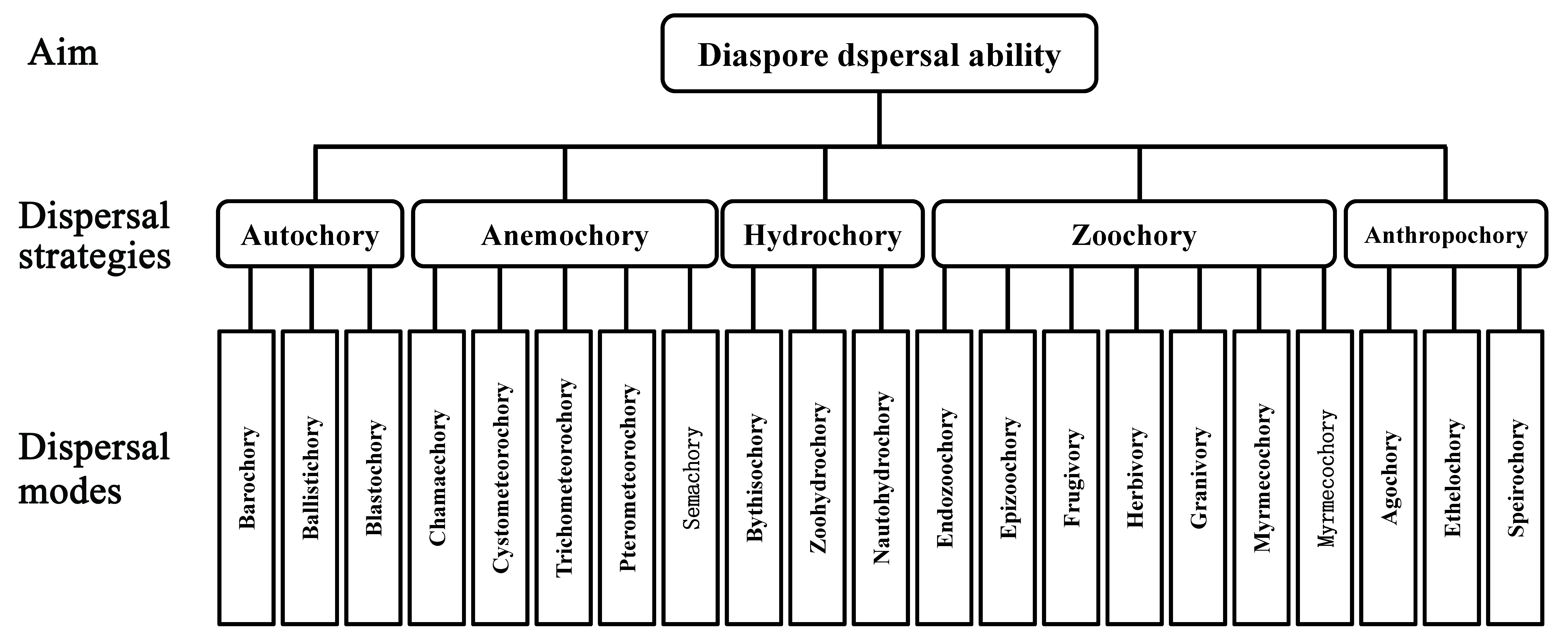


Fig. 2 Assessment indices system of diaspore dispersal ability

Step 2. Comparison of relative importance of the two dispersal strategies or dispersal modes according to dispersal distance of 21 dispersal modes (Table 1).

Table 1 Dispersal distances for 21 dispersal modes, estimated as the upper limits of the distances within which 50%and 99% of the diaspores of alien species are dispersed (van Oudtshoorn and van Rooyen 1999, Vittoz and Engler 2007).

| Dispersal distances | | Dispersal modes | Dispersal strategies |
| --- | --- | --- | --- |
| 50% | 99% |  |  |
| 0.1 | 1 | Blastochory | Autochory |
|  |  | Boleochory/ballochory for species < 30 cm | Anemochory |
|  |  | Barochory | Autochory |
| 1 | 5 | Ballochory | autochory |
|  |  | Cystometeorochory | anemochory |
|  |  | Chamaechory for fruits in grassland | anemochory |
|  |  | Boleochory for species > 30 cm | anemochory |
| 2 | 15 | Pterometeorochory for herbs | anemochory |
|  |  | Myrmecochory | zoochory |
|  |  | Cystometeorochory ferns, Orchidaceae, Pyrolaceae, Orobanchaceae in forest | anemochory |
|  |  | Trichometeorochory in forest or little efficient plumes | anemochory |
|  |  | Epizoochory for small mammals | zoochory |
|  |  | Herbivory | zoochory |
| 40 | 150 | Chamaechory for seeds on snow or dry inflorescence | anemochory |
|  |  | Pterometeorochory for trees | anemochory |
|  |  | Dyszoochory for seeds not stocked and dispersed by small animals | zoochory |
| 10 | 500 | Trichometeorochory in openland with efficient plumes | anemochory |
|  |  | Cystometeorochory ferns, Orchidaceae, Pyrolaceae, Orobanchaceae in openland | anemochory |
|  |  | Zoohydrochory | Hydrochory |
|  |  | Frugivory | zoochory |
|  |  | Granivory | zoochory |
| 400 | 1500 | Dyszoochory for seeds stocked by large animals | zoochory |
|  |  | Endozoochory for seeds eaten by birds and large vertebrates | zoochory |
|  |  | Epizoochory by large mammals | zoochory |
|  |  | Bythisochory | Hydrochory |
| 500 | 5000 | Agochory | anthropochory |
|  |  | Ethelochory | anthropochory |
|  |  | Speirochory | anthropochory |
|  |  | Nautohydrochory | Hydrochory |

Calculate the relative importance of two strategies or two modes using a numerical scale from 1 to 5 (Table 2). When first criterion compared to second criterion is assigned with a number between 1 and 5, this second criterion compared to first criterion becomes its reciprocal.

Table 2 Numerical scale for pairwise comparison

| Fuzzy linguistic variable | Numeric value |
| --- | --- |
| Extremely important | 5 |
| Very Strongly more important | 4 |
| Strongly more important | 3 |
| Moderately more important | 2 |
| Equally important | 1 |

Step 3. Generate pairwise comparison matrix of the dispersal strategies and modes in each strategy. The five strategies (dispersal agents) are autochory, anemochory, hydrochory, zoochory, anthropochory (Saaty 1979). Comparison matrix of the dispersal strategies is shown in Eq. (1).

$Dispersal strategies=\left( \begin{aligned} \begin{aligned} 1 \frac{1}{2} \frac{1}{2} \frac{1}{3} \frac{1}{4} \\ 2 1 \frac{1}{2} \frac{1}{3} \frac{1}{3} \end{aligned} \\ 2 2 1 \frac{1}{2} \frac{1}{3} \\ 3 3 2 1 \frac{1}{2} \\ 4 3 3 2 1 \end{aligned} \right)$ 1

Comparison matrix of the dispersal modes in autochory (barochory, ballistichory, and blastochory) is shown in Eq. (2).

$Dispersal modes in autochory=\left( \begin{aligned} 1 \frac{1}{3} \frac{1}{2} \\ 3 1 3 \\ 2 \frac{1}{3} 1 \end{aligned} \right)$ 2

Comparison matrix of the dispersal modes in anemochory (macrogeochory, cystometeorochory, trichometeorochory, and semachory) is shown in Eq. (3).

$Dispersal modes in \mathrm{anemochory}=\left( \begin{aligned} 1 \frac{1}{2} \frac{1}{3} 3 2 \\ 2 1 \frac{1}{3} 2 2 \\ 3 3 1 4 4 \\ \frac{1}{3} \frac{1}{2} \frac{1}{4} 1 2 \\ \frac{1}{2} \frac{1}{2} \frac{1}{4} \frac{1}{2} 1 \end{aligned} \right)$ 3

Comparison matrix of the dispersal modes in hydrochory (bythisochory, zoohydrochory, and nautohydrochory) is shown in Eq. (4).

$Dispersal modes in \mathrm{hydrochory}=\left( \begin{aligned} 1 \frac{1}{2} \frac{1}{3} \\ 2 1 1 \\ 3 1 1 \end{aligned} \right)$ 4

Comparison matrix of the dispersal modes in zoochory (endozoochory, epizoochory, frugivory, herbivory, granivory, myrmecochory and dyszoochory) is shown in Eq. (5).

$Dispersal modes in \mathrm{hydrochory}=\left( \begin{aligned} 1 3 2 2 4 3 2 \\ \frac{1}{3} 1 \frac{1}{3} 1 3 2 \frac{1}{2} \\ \frac{1}{2} 3 1 2 2 2 1 \\ \frac{1}{2} 1 \frac{1}{2} 1 2 1 2 \\ \frac{1}{4} \frac{1}{3} \frac{1}{2} \frac{1}{2} 1 \frac{1}{3} 1 \\ \frac{1}{3} \frac{1}{2} \frac{1}{2} 1 3 1 1 \\ \frac{1}{2} 2 1 \frac{1}{2} 1 1 1 \end{aligned} \right)$ 5

Comparison matrix of the dispersal modes in anthropochory (agochory, ethelochory, and speirochory) is shown in Eq. (6).

$Dispersal modes in \mathrm{hydrochory}=\left( \begin{aligned} 1 \frac{1}{2} 1 \\ 2 1 2 \\ 1 \frac{1}{2} 1 \end{aligned} \right)$ 6

Step 4. Normalize and check consistency of the comparison matrix (Eq. 1-6).

We use MATLAB software package to normalize the comparison matrix and to get the maximum eigenvalues of the matrix λ_max_. Consistency of a comparison matrix is tested by the following equation:

$CR=\frac{\mathrm{CI}}{\mathrm{RI}}=\frac{\frac{\lambda_{\max}-n}{n-1}}{\mathrm{RI}}$ 7

Where CI is the coincidence index; RI is the random coincidence index value (Table 2); n is the matrix order; and CR is the random coincidence coefficient, indicating the matrix satisfy the requirement of consistency check when the value of CR is smaller than 0.1 (Table 3).

Table 3 Random coincidence index value (RI)

| Content | Value | | | | | | | | |
| --- | --- | --- | --- | --- | --- | --- | --- | --- | --- |
| n | 1 | 2 | 3 | 4 | 5 | 6 | 7 | 8 | 9 |
| RI | 0 | 0 | 0.58 | 0.96 | 1.12 | 1.26 | 1.32 | 1.41 | 1.45 |

Step 5. Calculate dispersal strategies weight vector. We use MATLAB software package to calculate the weight vector of dispersal strategies (autochory, anemochory, hydrochory, zoochory, and anthropochory).

$W_{S}=\left( \begin{aligned} 0.0898 \\ 0.1708 \\ 0.0960 \\ 0.2678 \\ 0.3756 \end{aligned} \right)$ 8

Where W_S_ is the weight vector of dispersal strategies.

Step 6. Calculate dispersal modes weight vector in each strategy.

The weight vector of autochory (W_AUT_), anemochory (W_ANE_), hydrochory (W_HYD_), zoochory (W_ZOO_), and anthropochory(W_ANT_) are calculated as follows:

$W_{\mathrm{AUT}}=\left( \begin{aligned} 0.1571 \\ 0.5936 \\ 0.2493 \end{aligned} \right)$ 9

$W_{\mathrm{ANE}}=\left( \begin{aligned} 0.1708 \\ 0.2032 \\ 0.4408 \\ 0.1027 \\ 0.0825 \end{aligned} \right)$ 10

$W_{\mathrm{HYD}}=\left( \begin{aligned} 0.1692 \\ 0.3874 \\ 0.4434 \end{aligned} \right)$ 11

$W_{\mathrm{ZOO}}=\left( \begin{aligned} 0.2772 \\ 0.1158 \\ 0.1860 \\ 0.1277 \\ 0.0651 \\ 0.1062 \\ 0.1220 \end{aligned} \right)$ 12

$W_{\mathrm{ANT}}=\left( \begin{aligned} 0.25 \\ 0.50 \\ 0.25 \end{aligned} \right)$ 13

Step 7. Compute score vector (dispersal strategies weight vector × dispersal modes weight vector) (Table 4)

Table 4 Five dispersal categories and 21 dispersal modes of diaspores in alien species across China.

| Dispersal categories | Weight vector of dispersal strategy | Dispersal modes | | Weight vector of dispersal mode | Score vector for each dispersal mode |  |
| --- | --- | --- | --- | --- | --- | --- |
| Autochory | 0.0898 | 1 | Barochory | 0.1571 | 0.014108 | |
|  |  | 2 | Ballistichory | 0.5936 | 0.053305 | |
|  |  | 3 | Blastochory | 0.2493 | 0.022387 | |
| Anemochory | 0.1708 | 4 | Chamaechory | 0.1708 | 0.029173 | |
|  |  | 5 | Cystometeorochory | 0.2032 | 0.034707 | |
|  |  | 6 | Trichometeorochory | 0.4408 | 0.075289 | |
|  |  | 7 | Pterometeorochory | 0.1027 | 0.017541 | |
|  |  | 8 | Semachory | 0.0825 | 0.014091 | |
| Hydrochory | 0.096 | 9 | Bythisochory | 0.1692 | 0.016243 | |
|  |  | 10 | Zoohydrochory | 0.3874 | 0.03719 | |
|  |  | 11 | Nautohydrochory | 0.4434 | 0.042566 | |
| Zoochory | 0.2678 | 12 | Endozoochory | 0.2772 | 0.074234 | |
|  |  | 13 | Epizoochory | 0.1158 | 0.031011 | |
|  |  | 14 | Frugivory | 0.186 | 0.049811 | |
|  |  | 15 | Herbivory | 0.1277 | 0.034198 | |
|  |  | 16 | Granivory | 0.0651 | 0.017434 | |
|  |  | 17 | Myrmecochory | 0.1062 | 0.02844 | |
|  |  | 18 | Dyszoochory | 0.122 | 0.032672 | |
| Anthropochory | 0.3756 | 19 | Agochory | 0.25 | 0.0939 | |
|  |  | 20 | Ethelochory | 0.5 | 0.1878 | |
|  |  | 21 | Speirochory | 0.25 | 0.0939 | |

Step 8. Calculate diaspore dispersal ability. According to dispersal modes of each species, the dispersal ability of each species can be calculated as follows:

$DA=S_{1}+S_{2}+S_{3}+\ldots+S_{i}$ 14

Where DA is dispersal ability of each species; S is score of each dispersal mode; i is the ith dispersal mode.

**Reference**

Saaty, T. L. 1979. Applications of analytical hierarchies. Mathematics and Computers in Simulation **21**:1-20.

van Oudtshoorn, K. v. R. and M. W. van Rooyen. 1999. Dispersal biology of desert plants. Springer Berlin Heidelberg.

Vittoz, P. and R. Engler. 2007. Seed dispersal distances: a typology based on dispersal modes and plant traits. Botanica Helvetica **117**:109-124.
