# Supplementary material for: Geographical distribution of the dispersal ability of alien plant species in China and its socio-climatic control factors
Source: Sci Rep. 2021 Mar 30;11:7187. doi: 10.1038/s41598-021-85934-8 (PMC8009951; doi:10.1038/s41598-021-85934-8)
Supplement: Supplementary file 3 — Supplementary Material 3 [file 41598_2021_85934_MOESM3_ESM.docx]

**Geographical distribution of the dispersal ability of alien plant species in China and its socio-climatic control factors**

Quanlai Zhou^1, 2^, Jing Wu^3^, Xue Cui^4^, Xuehua Li^2^, Zhimin Liu^2^, Ala Musa^2^, Qun Ma^2^, Haibin Yu^2^, Wei Liang^2^, Shaoyan Jiang^5^, Yongcui Wang^1, 2*^

*^1^CAS Key Laboratory of Forest Ecology and Management, Institute of Applied Ecology, Chinese Academy of Sciences, Shenyang 110016, China*

*^2^Institute of Applied Ecology, Chinese Academy of Sciences, 72 Wenhua Road, Shenyang 110016, China*

*^3^Taizhou university, zhejiang Province, Taizhou, 384000, China*

*^4^Station of Forest and Grassland Pest Control and Quarantine of Liaoning Province, Shenyang 110804, China*

*^5^Liaoning Vocational College of Ecological Engineering, Shenyang 110101, China*

**Corresponding author: Yongcui Wang*

*Institute of Applied Ecology*

*Chinese Academy of Sciences*

*72 Wenhua Road, Shenyang, Liaoning Province 110016*

*Phone: (+86) 02483970431*

*Fax: (+86) 02483970300*

*Email:* [*yongcuiwang@iae.ac.cn*](mailto:yongcuiwang@iae.ac.cn)
